# Comparison of Mortality Risk Models in Patients with Postcardiac Arrest Cardiogenic Shock and Percutaneous Mechanical Circulatory Support

**DOI:** 10.1155/2021/8843935

**Published:** 2021-01-18

**Authors:** Georgios Chatzis, Birgit Markus, Styliani Syntila, Christian Waechter, Ulrich Luesebrink, Holger Ahrens, Dimitar Divchev, Bernhard Schieffer, Konstantinos Karatolios

**Affiliations:** Department of Cardiology, Angiology and Intensive Care, Philipps University Marburg, Marburg, Germany

## Abstract

**Background:**

Although scoring systems are widely used to predict outcomes in postcardiac arrest cardiogenic shock (CS) after out-of-hospital cardiac arrest (OHCA) complicating acute myocardial infarction (AMI), data concerning the accuracy of these scores to predict mortality of patients treated with Impella in this setting are lacking. Thus, we aimed to evaluate as well as to compare the prognostic accuracy of acute physiology and chronic health II (APACHE II), simplified acute physiology score II (SAPS II), sepsis-related organ failure assessment (SOFA), the intra-aortic balloon pump (IABP), CardShock, the prediction of cardiogenic shock outcome for AMI patients salvaged by VA-ECMO (ENCOURAGE), and the survival after venoarterial extracorporeal membrane oxygenation (SAVE) score in patients with OHCA refractory CS due to an AMI treated with Impella 2.5 or CP.

**Methods:**

Retrospective study of 65 consecutive Impella 2.5 and 32 CP patients treated in our cardiac arrest center from September 2015 until June 2020.

**Results:**

Overall survival to discharge was 44.3%. The expected mortality according to scores was SOFA 70%, SAPS II 90%, IABP shock 55%, CardShock 80%, APACHE II 85%, ENCOURAGE 50%, and SAVE score 70% in the 2.5 group; SOFA 70%, SAPS II 85%, IABP shock 55%, CardShock 80%, APACHE II 85%, ENCOURAGE 75%, and SAVE score 70% in the CP group. The ENCOURAGE score was the most effective predictive model of mortality outcome presenting a moderate area under the curve (AUC) of 0.79, followed by the CardShock, APACHE II, IABP, and SAPS score. These derived an AUC between 0.71 and 0.78. The SOFA and the SAVE scores failed to predict the outcome in this particular setting of refractory CS after OHCA due to an AMI.

**Conclusion:**

The available intensive care and newly developed CS scores offered only a moderate prognostic accuracy for outcomes in OHCA patients with refractory CS due to an AMI treated with Impella. A new score is needed in order to guide the therapy in these patients.

## 1. Introduction

Postcardiac arrest cardiogenic shock (CS) after out-of-hospital cardiac arrest (OHCA) complicating acute myocardial infarction (AMI) remains associated with a very poor prognosis, despite improvements in prehospital management and progress of postresuscitation care [1–3]. Ventricular failure subsequent to AMI remains the most frequent cause of cardiogenic shock (CS) accounting for more than 80% of cases [4]. In addition to inotropes, vasopressors, and revascularization of the infarct-related coronary artery, percutaneous left ventricular assist devices are used to support the circulation and improve the cardiac output and end-organ perfusion in these patients [5–7]. In a previous retrospective investigation, hemodynamic support with Impella was associated with improved survival compared to medical treatment in patients with postcardiac arrest CS related to AMI [8]. However, little is known about the management of patients treated with Impella in terms of survival prediction or survival with good neurological outcome. The most available scoring systems for survival after intensive care unit (ICU) admission, such as Acute Physiology and Chronic Health Score II (APACHE II), Simplified Acute Physiology Score II (SAPS II), and the sepsis-related organ failure assessment score (SOFA) have been used only sparsely in previous studies. However, none of them showed effective predictive value in patients with CS, especially in patients treated with percutaneous assist devices, such as the intra-aortic balloon pump (IABP) or Impella [9–12]. The IABP score is based on six parameters and was created in terms of the IABP Shock Trial to predict mortality in patients with CS undergoing support with IABP [13], while the CardShock score is also a relative newly developed score to predict outcomes in patients with heart failure [14]. These scores have been recently shown to be good predictors of in-hospital mortality in patients with CS; however, little is known about their predictive value in patients treated with Impella [12, 15, 16]. The prediction of cardiogenic shock outcome for AMI patients salvaged by VA-ECMO score (ENCOURAGE) and the survival after venoarterial extracorporeal membrane oxygenation (ECLS) score (SAVE) were originally developed for ECLS patients; there are only few studies that arranged the predictive value of these scores in patients treated exclusively with Impella [17–19].

In the same direction, the use of Impella in patients with severe CS is often related with adverse events and complications, demanding special training and resources. Therefore, more than ever, a reliable prediction model is needed in order to predefine which patients would benefit from the implantation of such a device. The patients with postcardiac arrest CS comprise a group of very ill patients with even worse outcome compared to patients without cardiac arrest. The implementation of predictive scores in these patients is a special challenge in the field of intensive care and cardiology. Since no study so far has focused on the predictive capacity of the aforementioned scores in postcardiac arrest patients due to an AMI treated with Impella, we aimed to study and compare the predictive accuracy of these scores in this setting of patients.

## 2. Methods

### 2.1. Patients' Characteristics

We retrospectively analyzed data from all patients resuscitated from OHCA due to AMI with postcardiac arrest CS who were supported with Impella 2.5 from September 2015 to June 2020. For this purpose, we reviewed our Impella registry to identify all OHCA patients admitted within this period to our institution who received Impella 2.5 or Impella CP support for postcardiac arrest CS complicating an AMI. All study patients underwent percutaneous coronary intervention (PCI). Patients with refractory OHCA under cardiopulmonary resuscitation on admission and patients with OHCA due to other causes were excluded from the analysis. Postcardiac arrest CS was defined as the need for continuous infusion of vasopressors to maintain systolic blood pressure >90 mmHg after return of spontaneous circulation. Timing of Impella implantation (pre- or post-PCI) was at the operating physician's discretion. Intention of therapy in cardiogenic shock was the use of Impella CP; however, Impella 2.5 was placed upon unavailability of this device. Coronary angiography and PCI were performed in a conventional manner. Patients were treated with drug-eluting stents and/or percutaneous transluminal coronary angioplasty. The extent of coronary revascularization and adjunctive therapies were left at the operator's discretion.

A subset (*n* = 27) of the Impella patients has been previously published [8]. The study was approved by the local ethics committee of the Philipps University of Marburg. The need for informed consent was waived due to the retrospective nature of the study. The study adheres to the STROBE guidelines for observational studies.

### 2.2. Device Management

All Impella devices were implanted through the femoral artery and placed via the retrograde approach through the aortic valve into the left ventricle under fluoroscopic control in the catheterization laboratory. All OHCA patients were treated with targeted temperature management (mild hypothermia of 33–34°C) for 24 h with an endovascular cooling device (Thermogard XP Temperature Management System, Zoll Medical Corporation, USA). After 24 h, gradual rewarming to 37°C in an hourly increment of 0.25°C was commenced in all patients. The intention was to maintain a body temperature below or equal to 37°C until 72 h after cardiac arrest. Inotropes and vasopressors were used to obtain a mean arterial pressure ≥65 mmHg. In patients with Impella support, flow was adjusted to maintain mean arterial pressure ≥65 mmHg with the lowest possible dose of catecholamines and to cover metabolic needs as assessed by central venous oxygen saturation (≥70%) and serum lactate levels (<2.0 mmol/L). A standardized protocol for the management of kidney function and the indication for renal replacement therapy was used. The decision to wean the circulatory support device was based on the resolution of shock and clinical assessment. Weaning process was performed by gradually decreasing Impella support. Once the support of the device was reduced to low levels (performance level 1) with stable mean arterial pressure ≥65 mmHg, no or low doses of catecholamines, central venous oxygen saturation ≥70%, and serum lactate levels <2.0 mmol/L, the device was removed in ICU, and hemostasis was achieved with a mechanical compression tool (FemoStop, Abbott Laboratories).

### 2.3. Data Collection and Study Endpoints

Intrahospital clinical data, outcomes, and follow-up data were collected from the medical charts. Prehospital arrest data were collected with the use of a preformatted standard data collection tool, including witnessed arrest, bystander CPR, no-flow time, duration of CPR, shockable or nonshockable rhythm, number of shocks, and epinephrine dosage during CPR.

The primary endpoint of our study was to assess the survival rates between patients supported with Impella 2.5 and CP as well as to compare the predictive value of SAPS II, SOFA, APACHE II, IABP shock, CardShock, ENCOURAGE, and SAVE score in these patients' collectives. Complication rates are also reported. Complications included bleeding at the insertion site, limb ischemia, and vascular complications requiring surgical or interventional repair. Bleeding was defined as blood loss at the Impella insertion site requiring blood transfusion, whereas other bleeding was defined as any bleeding irrespective of Impella use. Limb ischemia was defined as clinical hypoperfusion of the leg (decreased skin temperature of the leg and/or decreased peripheral pulses) requiring treatment or extraction of the device. Vascular complications requiring surgical or percutaneous repair were defined as intervention (surgical or percutaneous) on a vessel dissection, a pseudoaneurysm, an access-site thrombosis, or an arteriovenous fistula. Cerebral functional status was determined according to the Pittsburgh cerebral performance category (CPC) based on medical records or discharge summary abstracts.

### 2.4. Statistical Analysis

All data were analyzed retrospectively. Data are presented as absolute variables and percentages (%) for categorical variables and either median with interquartile range (IQR: 25^th^–75^th^ percentile) or mean with standard deviation according to the distribution of the variables. We assessed normality using Shapiro–Wilk test as well as Pearson test. After testing for normal distribution, Student's *t*-test or Mann–Whitney test was implemented to test for differences between various characteristics. For categorical variables, Fisher's exact test or chi-square test was used, as appropriate. In order to compare the predictive value of different scores, we calculated the area under the curve of each score.

All analyses were made using SPSS 24 (IBM Corp., USA) and GraphPad Prism 6.0. A two-sided *p* value <0.05 was considered statistically significant.

## 3. Results

From September 2015 to June 2020, a total of 97 consecutive patients who had postcardiac arrest CS related to AMI and underwent Impella implantation for left ventricle (LV) mechanical assistance and PCI were included in the present retrospective analysis. The 2.5 group consisted of 65 patients, whereas the group of CP consisted of 32 patients. The mean age of our Impella 2.5 cohort was 67.86 ± 12.82 years and of our Impella CP was 66 ± 13.92 years, with survivors being significantly younger (*p* < 0.001 in both groups), while 84.6% (55/65) in Impella 2.5 and 63% (20/32) in Impella CP were male without any difference in the distribution among survivors or nonsurvivors. All patients sustained an OHCA prior to admission, all patients were on mechanical ventilation, and all patients were in CS with need of catecholamines at the time of admission. On admission, median baseline systolic left ventricular ejection fraction (LVEF) was 32.19% ± 7.32% in the Impella 2.5 group showing no difference between survivors and nonsurvivors, whereas in the Impella CP group, the median LVEF was 31.07% ± 7.35%, showing a statistically significant difference between survivors and nonsurvivors. As expected, the age-adjusted Charlson comorbidity index was significantly lower among survivors in both groups, mainly driven by age and better kidney function. The nonsurvivors had a significantly worse cardiac arrest profile with longer periods of no-flow time, higher need for noradrenaline, and higher levels of renal clearance markers and serum lactate upon admission. In the group of Impella 2.5, 28 (43.1%) patients suffered from rib fractures and 14 (21.5%) from pneumothoraces that were successfully drained. In the Impella CP group, 14 patients (43.8%) were diagnosed with rib fractures upon admission, and 5 of them (14.7%) needed a pleural drainage for the pneumothorax. None of the traumatic injuries demanded further operative treatments. The baseline characteristics of the study population are listed in Table 1.

The device was successfully implanted through transfemoral access in all patients, while in 2 patients in the Impella 2.5 group, the PCI was not successful, one in the survivor group and one in the nonsurvivor group (*p*=1). A far as the procedural characteristics are concerned, there were no differences in the delays to hospital transfer or to balloon implantation (door to balloon) from the admission to the hospital. The duration of intervention and the amount of the contrast agent used were similar in both groups. The delay to Impella support was significantly lower among survivors in both groups compared to nonsurvivors (*p*=0.02 in 2.5 and *p*=0.03 in the CP group). In 14 (21.5%) patients of Impella 2.5 and in 9 (28.1%) patients of Impella CP, a multivessel intervention was successfully undertaken (no differences between the survivors and nonsurvivors in both groups). The extent of coronary artery disease was similar in both groups, and the culprit vessel was predominantly the left anterior descending artery. All procedural characteristics of the study population are demonstrated in Table 2.

The overall survival to hospital discharge in the entire cohort was 44.3% (43/97) (Table 3). The main cause of death was refractory CS occurring in 91.7% in the 2.5 group and in 83.3% in the CP group. Device-related vascular complications were more frequent among Impella CP patients (total of 16.9% and 28.1% in Impella 2.5 and CP, respectively). Access-site bleeding requiring transfusion occurred in 9.2% of the Impella 2.5 and in 21.9% of the Impella CP patients. Limb ischemia requiring extraction of the device and limb ischemia requiring percutaneous or surgical repair were also more frequent among the Impella CP patients. None of our patients experienced an in-hospital myocardial reinfarction, while one of the Impella CP patients suffered a stroke, leading to CPC 4 upon discharge. Nondevice-related bleeding was observed in 3 patients in the Impella 2.5 group and in 2 patients of Impella CP. All mentioned complication rates were comparable between the two study groups. The survival and safety outcomes are listed in Table 3.

**Figure 1:**
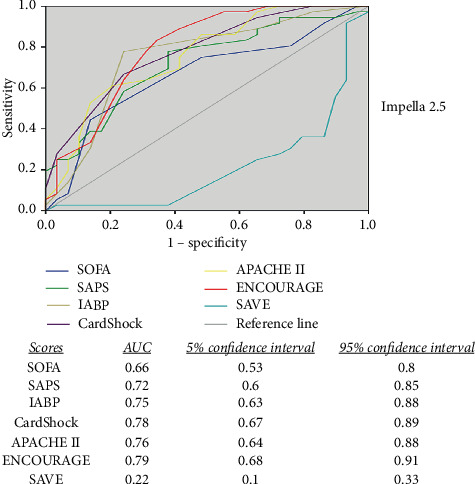
Comparison of the predictive values between the scores. Comparison of the sensitivity and specificity between different scores in patients supported with Impella 2.5. ENCOURAGE demonstrates the best area under the curve followed by CardShock, APACHE II, and IABP score. All scores demonstrate only a moderate prognostic accuracy; SAVE score cannot predict the outcome in this setting of patients.

**Figure 2:**
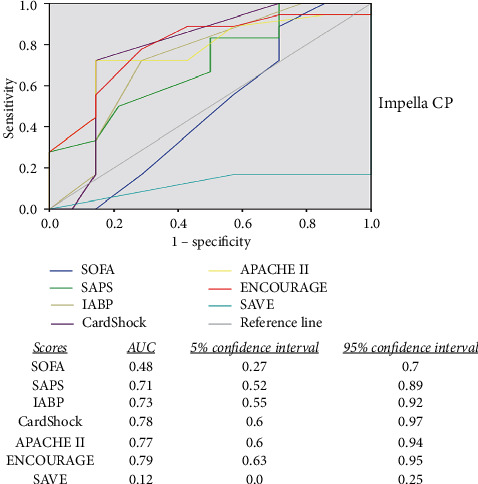
Comparison of the predictive values between the scores. Comparison of the sensitivity and specificity between different scores in patients supported with Impella CP. ENCOURAGE demonstrates the best area under the curve followed by CardShock, APACHE II, and IABP score. All scores demonstrate only a moderate prognostic accuracy; SAVE and SOFA scores cannot predict the outcome in these patients.

On admission, all ICU and CS scores were significantly higher in the group of the nonsurvivors (except for SOFA in the Impella CP group). According to the scores calculated on admission, it can be assumed that this patient group was critically ill; the expected mortality among Impella 2.5 patients according to SOFA, SAPS, IABP shock, CardShock, APACHE II, ENCOURAGE, and SAVE score was 70%, 90%, 55%, 80%, 85%, 50%, and 70%, respectively. Accordingly, the expected mortality of the Impella CP patients based on SOFA, SAPS, IABP shock, CardShock, APACHE II, ENCOURAGE, and SAVE score was 70%, 85%, 55%, 80%, 85%, 75%, and 70%, respectively. In our overall cohort, a total mortality rate of 55.2% was demonstrated, which represents a remarkable reduction according to the predicted mortality from the scores. The ENCOURAGE score appeared to be the most effective predictive model of mortality outcome in this setting of patients in both groups by reaching only a moderate AUC of 0.79, followed by the CardShock, APACHE II, IABP, and SAPS score. These derived an AUC between 0.71 and 0.78. The SOFA and the SAVE score did not appear to be effective predictors of the outcome in this particular setting of refractory CS after OHCA due to AMI. The predictive values of each score according to the groups are demonstrated in Figures 1 and 2.

## 4. Discussion

This analysis investigates, for the first time, solely patients supported with Impella in the particular setting of OHCA and postcardiac arrest CS complicating AMI in contrast to previous studies on mixed cohorts with and without previous cardiac arrest. To our knowledge, our analysis represents the largest single-center study to date concentrating on the use of Impella in OHCA patients with postcardiac arrest CS. The major finding of our study is the fact that the traditionally used prediction ICU and CS scores failed to offer a reliable prediction of outcome either in the setting of Impella 2.5 or CP LV unloading principle, while none of the scores reached an AUC of more than 0.8. On the contrary, it should be noted that the preimplantation cardiac arrest is a major determinant of CS mortality and that, in these patients, several factors quite rapidly influence 30-day mortality, which cannot solely be attributed to CS. For example, the anoxic brain death occurs before admission and cannot be influenced by the restoration of the hemodynamic profile from devices, such as Impella. This makes the development of scores in this patient collective very challenging. In our cohort, the best predictive performance was offered by the ENCOURAGE score, which consists of a score initially designed for ECMO patients, reaching only a moderate AUC of 0.79. Only few studies have questioned the predictive value of the most traditional and mostly used ICU and CS scores in patients treated with Impella for refractory CS. In the study by Sieweke et al., CardShock and IABP shock showed an acceptable predictive capacity among patients with CS [16]. However, in this study, only 60% of the participants were resuscitated prior to Impella implantation, and only 75% of the patients had CS due to an AMI. Moreover, all patients underwent an Impella CP implantation, which offers better and more effective unloading of the left ventricle accounting for better outcome. Under such perspectives, the assumption that these scores can effectively predict the outcome in OHCA with refractory CS due to a CS treated with Impella 2.5 remains premature. The study of all scores in this homogenous group of patients for both Impella devices is a major strength of our study. In another recent study of patients with CS due to an AMI, IABP score presented to have achieved a good predictive capacity in patients treated with Impella (true 20% survival in the high-risk group and 48% in the low-intermediate group, whereas score-predicted mortality was 80% and 50% in these groups, respectively) [12]. In this study, only 61% of the patients were resuscitated prior to Impella initiation, and only 20% of the participants had suffered non-ST-elevation myocardial infarction (NSTEMI). In the same study, the SAPS II score offered, with a median value of 68, an estimated mortality of almost 70%, clearly overestimated as compared to the final survival of 48%. Similarly, the SAPS II score overestimated the mortality in a group of 28 patients with profound CS treated with Impella CP (only 53.6% due to AMI, estimated mortality 87%, and true mortality 70%) [11]. In a study with biventricular unloading, the addition of Impella on ECLS in patients with severe LV dysfunction led to an improvement of the expected survival (SOFA score 12, estimated mortality more than 80%, and true survival 53%); however, only 52% of the patients had CS due to an AMI [9]. In the study by Schiller et al., the SAVE score offered only a moderate predictive value in patients with CS treated with Impella with a referred AUC of less than 0.65 [19]. In our cohort, ENCOURAGE score, though produced as the predictor for the outcome in ECLS patients, was the best predictor of mortality. There are several possible explanations for this finding. In the IABP and CardShock score, the cutoff point for scoring age is 73 and 75 years of age, respectively. This age is often seen as a contraindication for a mechanical support in patients suffering an OHCA so that the implementation of these scores could lead to underestimation of the predicted mortality due to the lack of weighting according to age. In the ENCOURAGE score, the cutoff value for age is 60 years. Moreover, the lactate values are the main contributor to the ENCOURAGE score: values >8 mmol/l result in 11 points in this scoring system, while more than 28 points represent the high-risk group. In comparison, the CardShock and IABP scores only give a maximum of 2 (out of 9) points for the highest lactate levels (cutoff >4 mmol/l and >5 mmol/l, respectively). However, the lactate levels are often very high in patients after successful CPR due to the dramatic and abrupt onset of diverse tissue hypoxia. The role of lactate as a determinant and predictor of outcome in patients after OHCA is undisputable [20, 21]. On the contrary, lactate is not utilized in SAPS, SOFA, APACHE II, and SAVE score. These latter scores appear to be attractive scores for the prediction of mortality in ICU since they are based on a broad spectrum of laboratory measurements, which depict the organ function. However, in patients after OHCA, these parameters are often useless since they are rarely changed or minimally elevated upon admission or directly after the initiation of CPR. The SAVE score presented the worst prediction model in our cohort of patients. This could be attributed to the fact that the cause of OHCA was an AMI and also that initiation of support was mediated directly after admission so that two of the main contributors in this score were without any fluctuation in this group of patients.

We describe an overall hospital mortality of 55.7% (54/97). The cause of death was refractory CS/multiorgan failure in 49.5% (48 patients: 33 from the Impella 2.5 group and 15 from the Impella CP group) and anoxic brain damage in 6.2% (6/97, 3 patients in each group) of the total patients. In the particular setting of OHCA survivors with postcardiac arrest CS, Manzo-Silberman and colleagues, comparing Impella with IABP, reported a lower survival rate of 23% at 28 days in the Impella group (and 29.5% in the IABP group, *p*=0.61) [7]. On the contrary, our survival rates are concordant to previous real-world Impella cohorts, including a mixed population of patients with and without prior cardiac arrest [22–25]. Compared to the randomized IABP-SHOCK II and IMPRESS trials, our survival rates were rather lower. However, we consider our Impella cohort to be at a higher risk for in-hospital death than the Impella patients enrolled in previous registries and the aforementioned randomized studies since all our patients were resuscitated before Impella support and were being mechanically ventilated, having a higher prevalence of nonshockable first rhythm, which is a traditional risk factor for a worse outcome [26–29]. Additionally, NSTEMI was present as a cause for the OHCA CS in 47.4% of the patients in our cohort in contrast to the randomized IMPRESS trial, which included only STEMI patients reporting a mortality of 46% at 30 days and 50% at 6 months. However, CS complicating NSTEMI has significantly higher mortality than CS complicating STEMI [30]. Survival rates in real-world cohorts are often lower than in randomized controlled trials with MCS. Our registry describes the clinical usage of Impella 2.5 and CP in an unselected cohort of patients in postcardiac arrest CS with greater risk features than those reported in randomized trials, reflecting higher mortality observed in routine clinical practice in such patients.

## 5. Study Limitations

There are several limitations to consider for our study. Firstly, the retrospective nature of this study limits a definitive causal relationship between the time of Impella placement and the survival outcome. Timing of Impella initiation, extent of revascularization, and adjunctive therapies were left to the operator's discretion and, therefore, subject to selection and treatment biases. Secondly, we could only retrieve adverse events and complications that were properly documented in the patients' chart, and we were not able to present the whole range of complications according to initial scores, which could also be interesting. We, therefore, focused on mortality outcomes as the primary endpoint, which were well documented in our Impella registry. Lastly, the relative small sample size is another limitation. However, our goal was to correlate the initial calculated scores with the final outcome as well as to calculate the predictive value of these scores. This is thought to be a unique intention till today. Moreover, we believe that the homogeneity of our population with all patients receiving Impella for support in the setting of OHCA with CS complicating AMI after successful PCI as well as the separate analysis among the groups of Impella CP and 2.5 offers a fair and direct comparison between the scores.

## 6. Conclusion

Although the predictive value of the score to an individual clinical situation will always remain a challenge, such scoring systems might ease the communication of objective prognostic information to family members and surrogate decision makers, help ICU physicians to identify severe AMI patients with reasonable chance of survival, and reduce futile healthcare. The traditionally used and the newly developed scores in the field of CS offer only moderate prognostic information in patients with cardiogenic shock after OHCA treated with Impella. A new more potent score is needed in this setting in order to guide clinicians and interventional cardiologists to optimize the therapy in this group of patients.

## Figures and Tables

**Table 1:** Demographics and baseline characteristics of the study population.

	Impella 2.5 (*N* = 65)	Survivors (*N* = 29)	Nonsurvivors (*N* = 36)	*p* value	Impella CP (*N* = 32)	Survivors (*N* = 14)	Nonsurvivors (*N* = 18)	*p* value
Age, years	67.86 ± 12.82	59.90 ± 12.23	74.28 ± 9.29	<0.001	66 ± 13.92	55.43 ± 13.30	74.22 ± 7.39	<0.001
Gender, male/female	55/10	25/4	30/6	1	20/12	10/4	10/8	0.47
BMI, kg/m^2^	27.60 ± 3.63	26.63 ± 2.91	28.35 ± 3.98	0.06	29.01 ± 3.33	27.37 ± 2.96	30.28 ± 3.09	0.01

*Medical comorbidities*								
Hypertension, *n* (%)	48 (73.8)	19 (65.5)	29 (80.6)	0.26	23 (72)	8 (57.1)	15 (83)	0.13
Diabetes, *n* (%)	21 (32.3)	9 (31)	12 (33.3)	1	16 (50)	6 (42.9)	10 (55.6)	0.72
PAD, *n* (%)	21 (32.3)	8 (27.6)	13 (36.1)	0.6	11 (34)	6 (42.9)	5 (27.8)	0.47
Stroke, *n* (%)	5 (7.7)	1 (3.3)	4 (11.1)	0.37	3 (9.4)	0 (0)	3 (16.7)	0.53
COPD, *n* (%)	12 (18.5)	4 (13.8)	8 (22.2)	0.52	7 (21.9)	2 (14.3)	5 (27.8)	0.43
Renal insufficiency (GFR <60 ml/min), *n* (%)	44 (67.7)	14 (48.3)	30 (83.3)	0.004	26 (81.3)	11 (78.6)	15 (83.3)	1
Prior CAD, *n* (%)	26 (40)	10 (34.5)	16 (44.4)	0.45	7 (21.9)	4 (28.6)	3 (16.7)	0.67
Prior MI, *n* (%)	23 (35.4)	9 (31)	12 (33.3)	1	5 (15.6)	2 (14.3)	3 (16.7)	1
Prior PCI, *n* (%)	22 (33.8)	9 (31)	13 (36.1)	0.79	2 (6.3)	2 (14.3)	0 (0)	0.18
Prior CABG, *n* (%)	9 (13.8)	3 (10.3)	6 (16.7)	0.72	2 (6.3)	2 (14.3)	0 (0)	0.18
Charlson comorbidity index (age-adjusted)	4.42 ± 2.93	2.64 ± 1.81	5.81 ± 2.9	<0.001	4.03 ± 2.82	2.86 ± 2.18	4.94 ± 2.98	<0.001

*Cardiac arrest variables*								
Witnessed arrest, *n* (%)	47 (72.3)	21 (72.4)	26 (72.2)	1	20 (62.5)	10 (71.4)	10 (55.6)	0.47
Shockable rhythm (VT or VF), *n* (%)	42 (64.6)	25 (86.2)	17 (47.2)	0.002	19 (59.4)	10 (71.4)	9 (50)	0.29
No-flow time (min)	4 [1.5–7]	2 [0–4.5]	6 [3–10]	0.0005	3.5 [2–6]	2 [1–4]	4.5 [3–10]	0.005
Low-flow time (min)	24.23 ± 13.86	19.03 ± 11.55	28.42 ± 14.29	0.0057	27.94 ± 15.58	23.71 ± 10.78	31.22 ± 18.11	0.18
Bystander CPR, *n* (%)	42 (64.6)	21 (72.4)	21 (58.3)	0.3	19 (59.4)	10 (71.4)	9 (50)	0.29
Number of electric shocks, median (IQR)	3 [1–5]	3 [2–6]	2.5 [0–4.75]	0.11	3 ± 2.72	4 ± 2.42	1 [0–5]	0.05
Epinephrine during resuscitation, *n* (%)	58 (89.2)	24 (82.8)	34 (94.4)	0.23	32 (100)	14 (100)	18 (100)	1
Total epinephrine dose during resuscitation (mg)	4 [1.9–6]	2 [1–5]	4 [2–6.5]	0.06	4 [3–6]	5 [3–6]	4 [2.75–6]	0.29
Time till ROSC (min)	28.71 ± 14.88	21.76 ± 11.74	34.31 ± 14.91	0.0005	28 [22.5–34]	27 [18–28]	33 [28–59]	0.008
Traumatic injuries on admission, *n* (%)	30 (46.2)	15 (51.7)	15 (41.7)	0.46	14 (43.8)	6 (42.9)	8 (44.4)	1

*Catecholamines*								
Inotropes (dobutamine), *n* (%)	51 (78.5)	25 (86.2)	26 (72.2)	0.23	24 (75)	12 (85.7)	12 (66.7)	0.41
Dobutamine (*μ*g/kg/min)	4.384 [0–6.41]	5.13 [3.39–6.31]	2.6 [0–7.1]	0.1	5.42 ± 1.6	5.4 ± 1.1	5.46 ± 2.03	0.97
Norepinephrine, *n* (%)	65 (100)	29 (100)	36 (100)	1	32 (100)	14 (100)	18 (100)	1
Norepinephrine (*μ*g/kg/min)	0.42 ± 0.25	0.33 ± 0.28	0.49 ± 0.21	0.01	0.36 ± 0.13	0.29 ± 0.16	0.41 ± 0.1	0.01
Epinephrine, *n* (%)	13 (20)	3 (10.3)	10 (27.8)	0.12	8 (25)	4 (28.6)	5 (27.8)	1
Epinephrine (*μ*g/kg/min)	0.12 ± 0.04	0.04 ± 0.03	0.18 ± 0.07	<0.001	0.65 ± 0.25	0.52 ± 0.19	0.76 ± 0.26	0.17
Mechanical ventilation, *n* (%)	65 (100)	29 (100)	36 (100)	1	32 (100)	14 (100)	18 (100)	1

*Hemodynamic variables on admission*								
Heart rate (bpm)	88.27 ± 22.27	81.43 ± 20.36	93.58 ± 22.51	0.03	98.31 ± 17.80	98.29 ± 19.55	98.33 ± 16.89	0.99
Systolic arterial pressure (mmHg)	97.44 ± 24.30	100.9 ± 24.42	94.75 ± 24.20	0.32	98.63 ± 13.30	93.29 ± 18.12	102.8 ± 5.48	0.04
Diastolic blood pressure (mmHg)	55.31 ± 13.71	57.86 ± 15.66	53.33 ± 11.83	0.19	58.47 ± 7.74	57.57 ± 10.65	59.17 ± 4.62	0.57
Mean blood pressure (mmHg)	69.35 ± 16.21	72.20 ± 17.37	67.14 ± 15.12	0.22	71.85 ± 8.85	69.48 ± 12.46	73.70 ± 3.98	0.18

*Blood values on admission*								
Lactate (mmol/L)	8.64 ± 3.96	5.89 ± 3.19	10.6 ± 3.57	<0.001	8.29 ± 2.49	6.77 ± 2.52	9.47 ± 1.75	0.001
Creatinine (mg/dl)	1.51 ± 0.50	1.24 ± 0.36	1.69 ± 0.5	0.0043	1.59 ± 0.53	1.33 ± 0.13	1.73 ± 0.62	0.05
GFR (ml/min)	49.26 ± 20.20	62.53 ± 20.63	40.61 ± 14.74	0.005	43.04 ± 14.06	51.56 ± 13.05	38.78 ± 12.83	0.03
Baseline LVEF (%)	32.19 ± 7.32	31.86 ± 7.55	32.35 ± 6.93	0.79	31.07 ± 7.35	34.45 ± 5.02	28.45 ± 7.92	0.02
STEMI at presentation, *n* (%)	35 (53.8)	19 (65.5)	16 (44.4)	0.13	16 (50)	10 (71.4)	6 (33.3)	0.07
Prehospital thrombolysis, *n* (%)	9 (13.8)	4 (13.8)	5 (13.9)	1	5 (15.6)	2 (14.3)	3 (16.7)	1
Duration of ICU stay, days	9.11 ± 7.23	14.5 ± 5.02	2 [1–6.75]	<0.001	11.25 ± 7.34	14.71 ± 6.32	8 [1–17]	0.05
Duration of hospital stay, days	9.97 ± 8.07	16.46 ± 5.37	2 [1–6.75]	<0.001	11.50 ± 7.54	15.29 ± 6.51	8 [1–17]	0.02

*Scores on admission*								
SAPS II	79.83 ± 7.83	76.66 ± 6.3	82.39 ± 8.08	0.003	79.5 ± 9.41	75.14 ± 10.36	82.89 ± 7.18	0.02
SOFA	11.57 ± 1.94	11.07 ± 2.1	11.97 ± 1.72	0.06	11.13 ± 2.41	10.86 ± 3.39	11.33 ± 1.28	0.59
IABP	3.59 ± 1.44	2.93 ± 1.39	4.11 ± 1.26	0.001	3.84 ± 1.22	3.36 ± 1.28	4.22 ± 1.06	0.04
APACHE II	33.17 ± 5.65	30.41 ± 5.34	35.39 ± 4.92	<0.001	34.84 ± 4.06	32.86 ± 3.11	36.39 ± 4.1	0.01
CardShock	6.4 ± 1.344	5.66 ± 1.29	7 ± 1.07	<0.001	6.56 ± 1.08	6.14 ± 1.35	6.9 ± 0.68	0.05
ENCOURAGE	25.46 ± 6.44	21.72 ± 6.6	28.47 ± 4.69	<0.001	27.63 ± 4.77	25.14 ± 4.13	29.56 ± 4.41	0.007
SAVE	−5.88 ± 4.94	−3.23 ± 4.35	−7.97 ± 4.39	<0.001	−7 ± 3.61	−4.29 ± 1.82	−9.11 ± 3.23	<0.001

BMI: body mass index; PAD: peripheral artery disease; COPD: chronic obstructive pulmonary disease; GFR: glomerular filtration rate; PCI: percutaneous coronary intervention; CABG: coronary artery bypass graft; VT: ventricular tachycardia; VF: ventricular fibrillation; CPR: cardiopulmonary resuscitation; ROSC: return of spontaneous circulation; LVEF: left ventricular ejection fraction; STEMI: ST-elevation myocardial infarction; ICU: intensive care unit; SAPS II: simplified acute physiology score II; SOFA: sequential organ failure assessment; IABP: intra-aortic balloon pump; APACHE: acute physiology and chronic health; ENCOURAGE: prediction of cardiogenic shock outcome for AMI patients salvaged by VA-ECMO; SAVE: survival after venoarterial extracorporeal membrane oxygenation (VA-ECMO). Numbers are presented as mean (±standard deviation) or median (interquartile range, IQR 25^th^–75^th^ percentile) or frequency (percentile).

**Table 2:** Procedural characteristics of the overall cohort.

	All patients (*n* = 65)	Survivors (*n* = 29)	Nonsurvivors (*n* = 36)	*p* value	Impella CP (*N* = 32)	Survivors (*N* = 14)	Nonsurvivors (*N* = 18)	*p* value
Duration of Impella support (hours)	71 [14–127.5]	118.5 [69.25–144]	31 [6.5–79.5]	0.0003	78.77 ± 52.53	105 ± 44.44	48 [12–88.5]	0.04
Door to balloon (min)	85.21 ± 39.47	86.63 ± 40.24	83.90 ± 39.4	0.8	73 [69–102]	73 [65–79]	73 [69–124]	0.37
Door to Impella support (min)	105.5 ± 53.48	87 ± 42.78	120.9 ± 57.18	0.02	72.5 [57.5–105]	62.5 [43–80]	102.5 [61–150.3]	0.03
Time from ROSC to hospital admission (min)	75.98 ± 36	74.58 ± 37.69	77.06 ± 35.23	0.8	56.83 ± 34.54	57.44 ± 24.90	56.38 ± 41.13	0.93
Number of stents used^*∗*^	2 [1–3]	1.5 [1–3]	2 [1–3]	0.99	3 [1–3.75]	3 [1–4]	2 [1–3]	0.35

*Culprit vessel*, *n* (%)								
Left main	3 (4.6)	1 (3.4)	2 (5.6)	NS for all comparisons	2 (6.3)	2 (14.3)	0 (0)	NS for all comparisons
LAD	35 (53.8)	17 (58.6)	18 (50)		16 (50)	7 (50)	9 (50)	
LCx	13 (20)	7 (24.1)	6 (16.7)		5 (15.6)	2 (14.3)	3 (16.7)	
RCA	12 (18.5)	4 (13.9)	8 (22.2)		6 (18.8)	3 (21.4)	3 (16.7)	
Bypass graft	2 (3.1)	0 (0)	2 (5.5)		3 (9.3)	0 (0)	3 (16.7)	

*Number of vessels diseased* ^*∗∗*^								
1	15 (23.1)	9 (31)	6 (16.7)	NS for all comparisons	5 (15.6)	2 (14.3)	3 (16.7)	NS for all comparisons
2	20 (30.8)	8 (27.6)	12 (33.3)		11 (34.4)	4 (28.6)	7 (38.9)	
3	30 (46.2)	12 (41.4)	18 (50)		16 (50)	8 (57.1)	8 (44.4)	

Multivessel intervention	14 (21.5)	8 (27.6)	6 (16.7)	0.37	9 (28.1)	4 (28.6)	5 (27.8)	0.45
Successful PCI	63 (96.9)	28 (96.5)	35 (97.2)	1	32 (100)	14 (100)	18 (100)	1

*Use of GP IIb/IIIa receptor inhibitors*								
Tirofiban	9 (13.8)	7 (24.1)	2 (5.5)	NS for all comparisons	6 (18.8)	4 (28.6)	2 (11.1)	NS for all comparisons
Abciximab	6 (9.2)	1 (3.4)	5 (13.9)		3 (9.4)	3 (21.4)	0 (0)	

Duration of intervention (min)	119 ± 47.01	112.2 ± 57.09	124.5 ± 37.07	0.33	125.3 ± 50.95	128.0 ± 68.05	122.9 ± 31.51	0.79
Contrast agent (ml)	289.4 ± 122.4	308.3 ± 143.7	273.8 ± 101.6	0.31	347 ± 150	375.7 ± 173.9	321.9 ± 125.9	0.34

LAD: left anterior descending artery, LCx: left circumflex artery, RCA: right coronary artery, ROSC: return of spontaneous circulation, PCI: percutaneous coronary intervention; GP: glycoprotein. Numbers are presented as mean (± standard deviation), median (interquartile range, IQR 25th–75th percentile) or frequency (percentile). ^*∗*^Only drug-eluting stents were used. ^*∗∗*^>50% stenosis in the nonculprit vessel.

**Table 3:** Survival and safety outcomes.

	Impella 2.5 (*n* = 65)	Survivors (*n* = 29)	Nonsurvivors (*n* = 36)	*p* value	Impella CP (*N* = 32)	Survivors (*N* = 14)	Nonsurvivors (*N* = 18)	*p* value
Survival to hospital discharge, *n* (%)	29 (44.6)	29 (100)	—	—	14 (43.8)	14 (100)	—	—
CPC 1–2, *n* (%)	22 (75.9)	22 (75.9)	—	—	11 (78.6)	11 (78.6)	—	—
CPC 3–4, *n* (%)	7 (24.1)	7 (24.1)	—	—	3 (21.4)	3 (21.4)	—	—

*Causes of death*								
Cardiogenic shock/MOF, *n* (%)	33 (50.8)	—	33 (91.7)	—	—	—	15 (83.3)	—
Brain death, *n* (%)	3 (4.6)	—	3 (8.3)	—	—	—	3 (16.7)	—

*Complications*								
Access-site bleeding requiring transfusion, *n* (%)	6 (9.2)	3 (10.3)	3 (8.3)	1	7 (21.9)	3 (21.4)	4 (22.2)	1
Limb ischemia requiring extraction of the device, *n* (%)	3 (4.6)	1 (3.4)	2 (5.6)	1	5 (15.7)	2 (14.3)	3 (16.7)	1
Limb ischemia requiring intervention, *n* (%)	2 (3.1)	1 (3.4)	1 (2.8)	1	4 (12.5)	2 (14.3)	2 (11.1)	1
Pericardial effusion needing paracentesis, *n* (%)	1 (1.5)	1 (2.5)	0 (0)	1	1 (3.1)	0 (0)	1 (5.6)	1
Myocardial reinfarction, *n* (%)	0 (0)	0 (0)	0 (0)	1	0 (0)	0 (0)	0 (0)	1
Stroke, *n* (%)	0 (0)	0 (0)	0 (0)	1	1 (3.1)	0 (0)	1 (5.6)	1
Nondevice-related bleeding, *n* (%)	3 (4.6)	1 (3.6)	2 (5.6)	1	2 (6.3)	1 (7.1)	1 (5.6)	1

CPC: cerebral performance category; MOF: multiorgan failure. Numbers are presented as frequencies (percentile).

## Data Availability

The data used to support the findings of this study are available from the corresponding author upon reasonable request.

## References

[1] Benjamin E. J., Blaha M. J., Chiuve S. E. (2017). Heart disease and stroke statistics-2017 update: a report from the American heart association. *Circulation*.

[2] Girotra S., Nallamothu B. K., Spertus J. A., Li Y., Krumholz H. M., Chan P. S. (2012). Trends in survival after in-hospital cardiac arrest. *New England Journal of Medicine*.

[3] Berdowski J., Berg R. A., Tijssen J. G. P., Koster R. W. (2010). Global incidences of out-of-hospital cardiac arrest and survival rates: systematic review of 67 prospective studies. *Resuscitation*.

[4] Hochman J. S., Buller C. E., Sleeper L. A. (2000). Cardiogenic shock complicating acute myocardial infarction--etiologies, management and outcome: a report from the SHOCK trial registry should we emergently revascularize occluded coronaries for cardiogenic shock?. *Journal of the American College of Cardiology*.

[5] Thiele H., Ohman E. M., de Waha-Thiele S., Zeymer U., Desch S. (2019). Management of cardiogenic shock complicating myocardial infarction: an update 2019. *European Heart Journal*.

[6] de Chambrun M. P., Bréchot N., Lebreton G. (2016). Venoarterial extracorporeal membrane oxygenation for refractory cardiogenic shock post-cardiac arrest. *Intensive Care Medicine*.

[7] Manzo-Silberman S., Fichet J., Mathonnet A. (2013). Percutaneous left ventricular assistance in post cardiac arrest shock: comparison of intra aortic blood pump and IMPELLA recover LP2.5. *Resuscitation*.

[8] Karatolios K., Chatzis G., Markus B. (2018). Impella support compared to medical treatment for post-cardiac arrest shock after out of hospital cardiac arrest. *Resuscitation*.

[9] Colombier S., Quessard A., Mastroianni C. (2019). Benefits of impella and peripheral veno-arterial extra corporeal life support alliance. *ASAIO Journal*.

[10] Gaudard P., Mourad M., Eliet J. (2015). Management and outcome of patients supported with Impella 5.0 for refractory cardiogenic shock. *Critical Care*.

[11] Lackermair K., Sattler S., Huber B. C. (2016). Retrospective analysis of circulatory support with the impella CP device in patients with therapy refractory cardiogenic shock. *International Journal of Cardiology*.

[12] Alushi B., Douedari A., Froehlig G. (2019). Impella versus IABP in acute myocardial infarction complicated by cardiogenic shock. *Open Heart*.

[13] Pöss J., Köster J., Fuernau G. (2017). Risk stratification for patients in cardiogenic shock after acute myocardial infarction. *Journal of the American College of Cardiology*.

[14] Harjola V.-P., Lassus J., Sionis A. (2015). Clinical picture and risk prediction of short-term mortality in cardiogenic shock. *European Journal of Heart Failure*.

[15] Rivas-Lasarte M., Sans-Rosello J., Collado-Lledo E. (2020). External validation and comparison of the CardShock and IABP-SHOCK II risk scores in real-world cardiogenic shock patients. *European Heart Journal: Acute Cardiovascular Care*.

[16] Sieweke J. T., Berliner D., Tongers J. (2020). Mortality in patients with cardiogenic shock treated with the Impella CP microaxial pump for isolated left ventricular failure. *European Heart Journal: Acute Cardiovascular Care*.

[17] Muller G., Flecher E., Lebreton G. (2016). The ENCOURAGE mortality risk score and analysis of long-term outcomes after VA-ECMO for acute myocardial infarction with cardiogenic shock. *Intensive Care Medicine*.

[18] Chen W. C., Huang K. Y., Yao C. W. (2016). The modified SAVE score: predicting survival using urgent veno-arterial extracorporeal membrane oxygenation within 24 hours of arrival at the emergency department. *Critical Care*.

[19] Schiller P., Hellgren L., Vikholm P. (2019). Survival after refractory cardiogenic shock is comparable in patients with impella and veno-arterial extracorporeal membrane oxygenation when adjusted for SAVE score. *European Heart Journal: Acute Cardiovascular Care*.

[20] Lee D. H., Cho I. S., Lee S. H. (2015). Correlation between initial serum levels of lactate after return of spontaneous circulation and survival and neurological outcomes in patients who undergo therapeutic hypothermia after cardiac arrest. *Resuscitation*.

[21] Adrie C., Cariou A., Mourvillier B. (2006). Predicting survival with good neurological recovery at hospital admission after successful resuscitation of out-of-hospital cardiac arrest: the OHCA score. *European Heart Journal*.

[22] Lauten A., Engström A. E., Jung C. (2013). Percutaneous left-ventricular support with the impella-2.5-assist device in acute cardiogenic shock. *Circulation: Heart Failure*.

[23] Loehn T., O’Neill W. W., Lange B. (2020). Long term survival after early unloading with Impella CP^®^ in acute myocardial infarction complicated by cardiogenic shock. *European Heart Journal: Acute Cardiovascular Care*.

[24] O’Neill W. W., Schreiber T., Wohns D. H. W. (2014). The current use of impella 2.5 in acute myocardial infarction complicated by cardiogenic shock: results from the USpella registry. *Journal of Interventional Cardiology*.

[25] Ouweneel D. M., de Brabander J., Karami M. (2019). Real-life use of left ventricular circulatory support with Impella in cardiogenic shock after acute myocardial infarction: 12 years AMC experience. *European Heart Journal: Acute Cardiovascular Care*.

[26] Wibrandt I., Norsted K., Schmidt H. (2015). Predictors for outcome among cardiac arrest patients: the importance of initial cardiac arrest rhythm versus time to return of spontaneous circulation, a retrospective cohort study. *BMC Emergency Medicine*.

[27] Martinell L., Nielsen N., Herlitz J. (2017). Early predictors of poor outcome after out-of-hospital cardiac arrest. *Critical Care*.

[28] Herlitz J., Engdahl J., Svensson L., Ängquist K.-A., Young M., Holmberg S. (2005). Factors associated with an increased chance of survival among patients suffering from an out-of-hospital cardiac arrest in a national perspective in Sweden. *American Heart Journal*.

[29] Holmberg M., Holmberg S., Herlitz J. (2000). Incidence, duration and survival of ventricular fibrillation in out-of-hospital cardiac arrest patients in Sweden. *Resuscitation*.

[30] Anderson M. L., Peterson E. D., Peng S. A. (2013). Differences in the profile, treatment, and prognosis of patients with cardiogenic shock by myocardial infarction classification. *Circulation: Cardiovascular Quality and Outcomes*.

